# A Rare Case of a Large Vulvovaginal Cellular Leiomyoma in a Young Female Patient: A Case Report

**DOI:** 10.7759/cureus.117919

**Published:** 2026-10-14

**Authors:** Elene Lipartia, Tamaz Nikolaishvili, David Gagua, Tinatin Gagua, Beka Metreveli, Nino Demetrashvili, Aleksandre Gvaladze

**Affiliations:** 1 Obstetrics and Gynaecology, Gagua Clinic, Tbilisi, GEO; 2 Obstetrics and Gynaecology, David Tvildiani Medical University, Gagua Clinic, Tbilisi, GEO

**Keywords:** benign mesenchymal tumor, cellular leiomyoma, desmin expression, h-caldesmon, ki67, large vulvovaginal mass

## Abstract

We present a rare clinical case of a 40-year-old woman with slowly growing vulvovaginal masses. According to the patient's history, the tumors had been gradually increasing in size over the past six years. Physical examination revealed two independent, well-circumscribed masses: a large mass in the left vulvar region and a medium-sized mass in the anterior vaginal wall. Magnetic resonance imaging (MRI) showed that both lesions were of soft tissue origin and did not invade adjacent organs. Histomorphological examination of the incisional biopsy revealed a benign mesenchymal neoplasia with signs of smooth muscle differentiation, increased cellularity, without cytological atypia, pronounced mitotic activity, and tumor necrosis.

After radical surgical excision, the diagnosis of cellular leiomyoma was confirmed. Reconstructive surgery was performed to correct the defect resulting from the removal of the large vulvar mass. The postoperative period was uneventful, and the patient was discharged on the first day after surgery.

This case highlights the diagnostic difficulty of rare vulvovaginal mesenchymal tumors and demonstrates the importance of a comprehensive clinical, radiological, and histopathological evaluation for accurate diagnosis and appropriate treatment.

## Introduction

Vulvar leiomyoma is a rare benign smooth muscle tumor that is often challenging to diagnose. Due to its rare location and nonspecific clinical presentation, vulvar leiomyoma can often be misdiagnosed preoperatively as a more common vulvar mass, including Bartholin's cyst, lipoma, or malignant soft tissue tumor. Although vulvar leiomyoma can occur in women of all ages, it is most commonly diagnosed in women of reproductive age, with a reported median age of approximately 41 years [1]. The cellular variant is a rare histological subtype of leiomyoma that is more commonly found in the uterus and is characterized by the presence of densely packed, highly cellular, and spiked smooth muscle cells.

Extrauterine localization, including vaginal and vulvar origin, is extremely rare. Among leiomyomas of the soft tissue of the female genital tract, vaginal localization is more common than vulvar, with a ratio of approximately 3:1. Vaginal leiomyomas can develop in the upper, middle, or lower third of the vagina, while vulvar leiomyomas are more often described in the mons pubis and labia majora, and relatively rarely in the labia minora [1-3].

The high cellularity of this variant may pose diagnostic difficulties, as it may morphologically resemble malignant smooth muscle tumor or other vulvovaginal mesenchymal neoplasms. Therefore, histopathological evaluation and correlation with immunohistochemical data are necessary for an accurate diagnosis. Despite its high cellularity, the prognosis of benign cellular leiomyoma is very good, and transformation to vulvar leiomyosarcoma is considered an extremely rare event, with the reported risk usually being less than 1% [2,3]. The mainstay of treatment remains complete surgical excision. A definitive diagnosis usually requires histopathological evaluation of surgically excised material, as accurate differentiation between benign and malignant tumors is often not possible based on clinical and imaging findings alone. In selected cases, especially in vaginal tumors, diagnostic core biopsy may be used.

The present case describes a large vulvar mesenchymal mass in a young patient, whose final morphological evaluation confirmed the diagnosis of cellular leiomyoma. The case highlights the importance of an accurate differential diagnosis of vulvovaginal mesenchymal tumors and the need for an individualized treatment approach.

## Case presentation

A woman in her 40s presented to the gynecology clinic with a complaint of a vulvovaginal mass. According to the patient, the lesion had been slowly increasing in size over the preceding six years. She had not sought medical attention earlier due to fear that the mass might represent a malignant process. Her past medical history was significant for pelvic inflammatory disease complicated by sepsis, which necessitated a total hysterectomy at the age of 25 years. Additionally, she had undergone surgical excision of a benign vulvar mass approximately 10 years prior to the current presentation.

Gynecological examination revealed the presence of two distinct tumors. The larger mass presented as a marked enlargement of the left labium, extending from the clitoral region anteriorly to the perineal body and anus. On palpation, the lesion was firm, non-tender, and well-circumscribed; however, assessment of its overall mobility was limited due to its size. Rectal examination demonstrated free mobility of the rectal mucosa over the posterior surface of the mass, suggesting the absence of rectal wall invasion. Vaginal examination was unable to fully delineate the posterior extent of the lesion because of its size. The gross dimensions of the larger tumor were approximately 15-20 cm (Figure 1).

**Figure 1:**
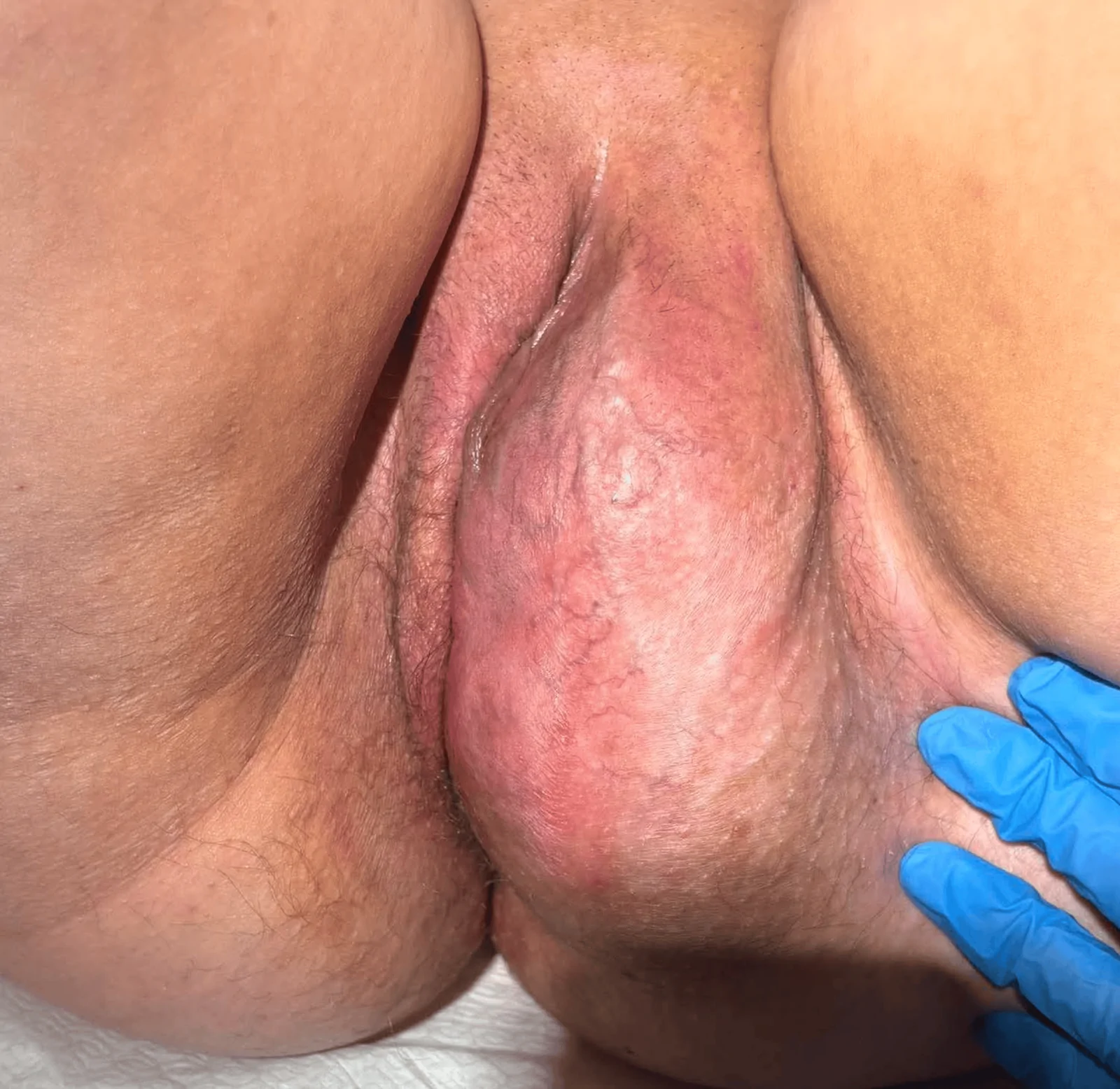
Preoperative clinical picture of a vulvar mass A well-circumscribed, large soft-tissue mass is seen arising from the left side of the vulva. The lesion appears exophytic and localized, with no obvious overlying skin ulceration or active bleeding. The surrounding vulvar architecture is partially distorted due to the size of the tumor.

A second, smaller mass was identified within the anterior vaginal wall. This lesion was smooth, well-defined, and freely mobile over the underlying bladder wall, with no clinical evidence of local invasion. Perineal and transvaginal ultrasonography demonstrated that both lesions were solid fibrous tumors measuring 14.2x9.3x5.7 cm and 5,2x4,5x3 cm, respectively. Dynamic ultrasound examination confirmed that the smaller lesion moved freely relative to the bladder wall, further supporting the absence of bladder involvement. Given the substantial size of the larger tumor and the inability to adequately define its anatomical boundaries on clinical examination and ultrasonography, pelvic magnetic resonance imaging (MRI) was performed for further characterization and preoperative assessment. Magnetic resonance imaging (MRI) demonstrated two distinct soft-tissue masses and post-hysterectomy changes. In the postoperative area, a rounded, well-defined, actively enhancing soft-tissue mass, measuring up to 7 mm, is visualized. The described lesion is not associated with deep anterior invasion; it is closely related to the dorsal wall of the urinary bladder and causes its compression and deviation. Caudally from the above-mentioned lesion, a markedly heterogeneous, polycyclic mass is seen. It cannot be clearly differentiated from the vaginal walls, vulvar structures, or the levator ani muscles. The lesion consists of several components, including fibrotic, microcystic, and solid soft-tissue components, with active contrast enhancement. It has a conglomerate appearance and measures up to 17 cm (Figures 2A-2D).

**Figure 2:**
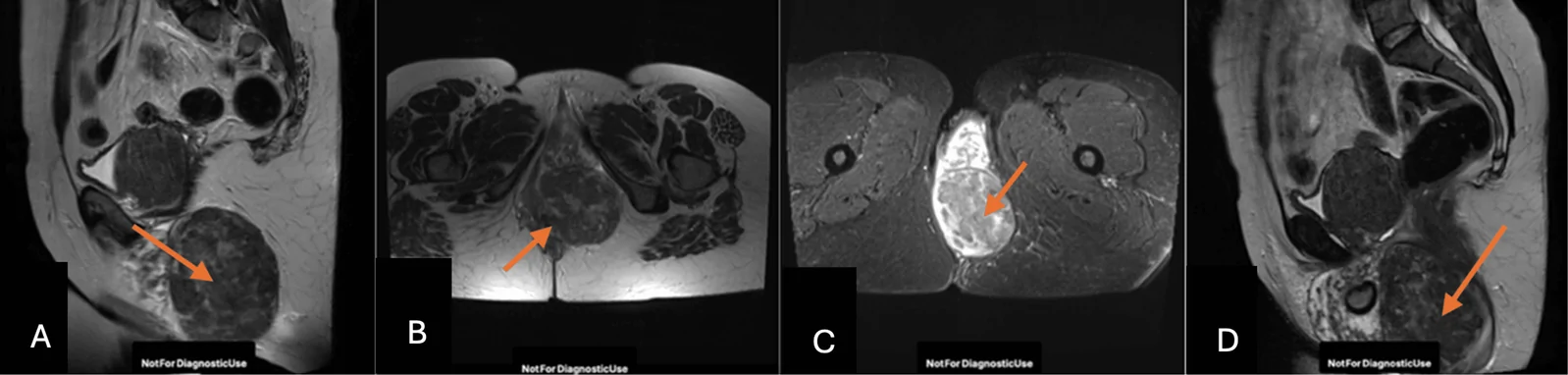
Pelvic MRI (A-D) Pelvic MRI demonstrating a large heterogeneous soft-tissue mass measuring approximately 17 cm, with fibrotic, microcystic, and solid components and heterogeneous contrast enhancement. The mass extends into the perineal region and causes marked compression and displacement of the urinary bladder and rectum, without definite evidence of deep invasion.

Despite its size and close relationship to surrounding structures, there was no radiological evidence of intra-abdominal extension or invasion of adjacent pelvic organs.

An incisional biopsy was obtained from the larger tumor located along the lateral vaginal wall (Figure 3). Gross examination revealed multiple fragments of gray-white, soft tissue measuring 1.3 × 0.8 × 0.6 cm in aggregate. Histopathological examination demonstrated a benign mesenchymal neoplasm composed of spindle-shaped cells arranged in parallel fascicles. The cells exhibited elongated, oval nuclei with finely dispersed chromatin and abundant eosinophilic cytoplasm. Focal myxoid stromal areas were present throughout the lesion. No significant cytologic atypia, tumor cell necrosis, or increased mitotic activity was identified. Based on the histomorphological findings, the lesion was diagnosed as benign mesenchymal proliferation.

**Figure 3:**
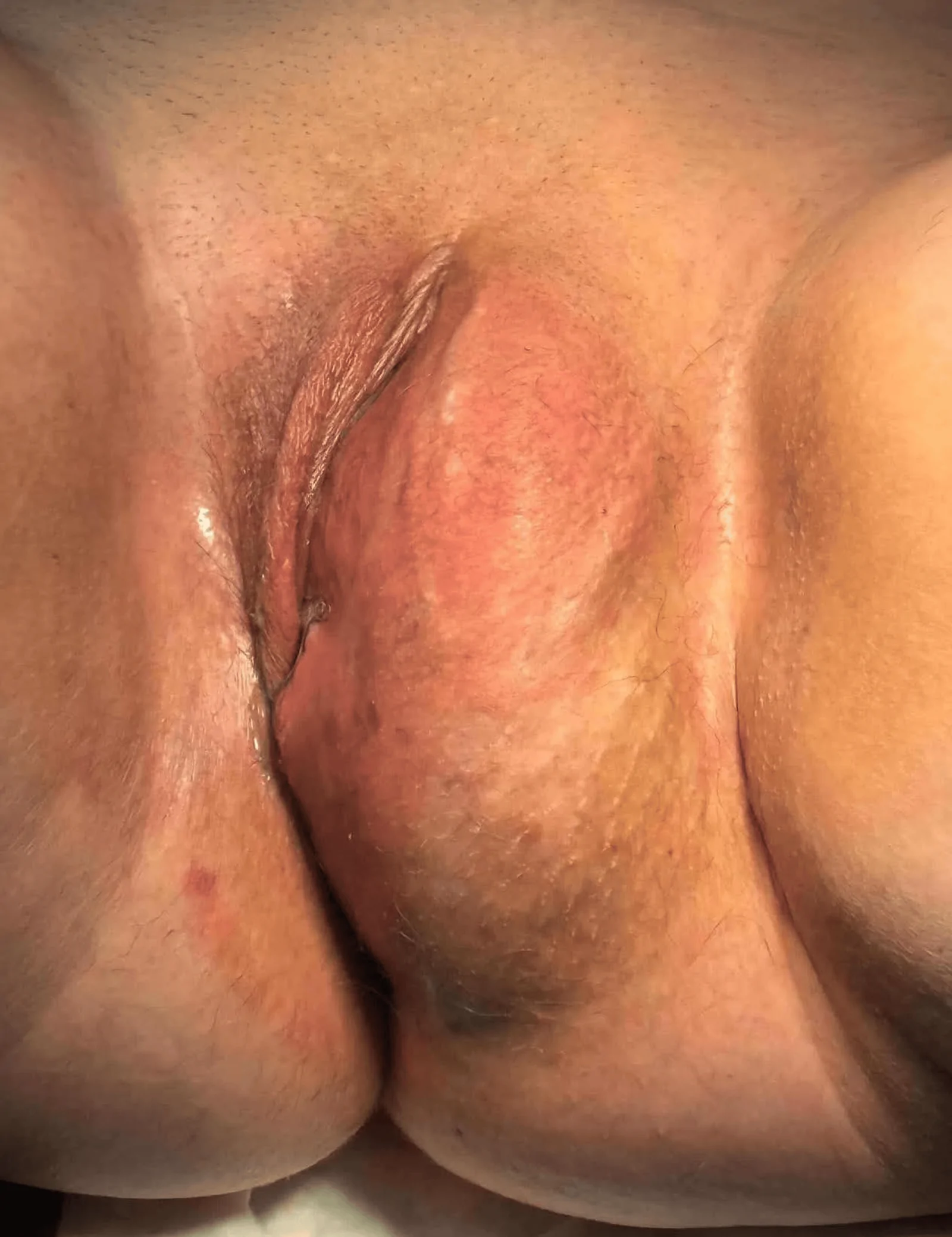
Post-biopsy appearance of the vulvar mass The left vulvar mass following biopsy demonstrates a localized biopsy site with minimal surface disruption. There is no active bleeding or signs of acute infection. The lesion remains grossly unchanged in size.

Surgical treatment

Surgical excision was performed one month after the initial examination. At the time of surgery, both tumors remained unchanged in size and clinical characteristics. Preoperative assessment revealed no evidence of rectal or urinary bladder involvement. The procedure was carried out under spinal anesthesia. For excision of the larger mass, a vertical incision measuring approximately 10 cm was made over the vaginal aspect of the lesion. Following careful sharp and blunt dissection, the tumor margins were easily identified. The majority of the mass was mobilized and excised using predominantly blunt dissection techniques (Figures 4A-4H). Feeding vessels encountered during the procedure were individually identified, clamped, and ligated. The inferior portion of the tumor, located in close proximity to the rectum, was meticulously dissected and excised under digital rectal guidance to minimize the risk of rectal injury. Intraoperative blood loss was moderate. Following tumor removal, a substantial soft-tissue defect and redundant tissue remained. Therefore, reconstructive vaginoplasty was performed. Closure was achieved in three layers using absorbable polyglactin sutures, while a drainage opening of approximately 2 cm was intentionally left at the most dependent portion of the wound.

**Figure 4:**
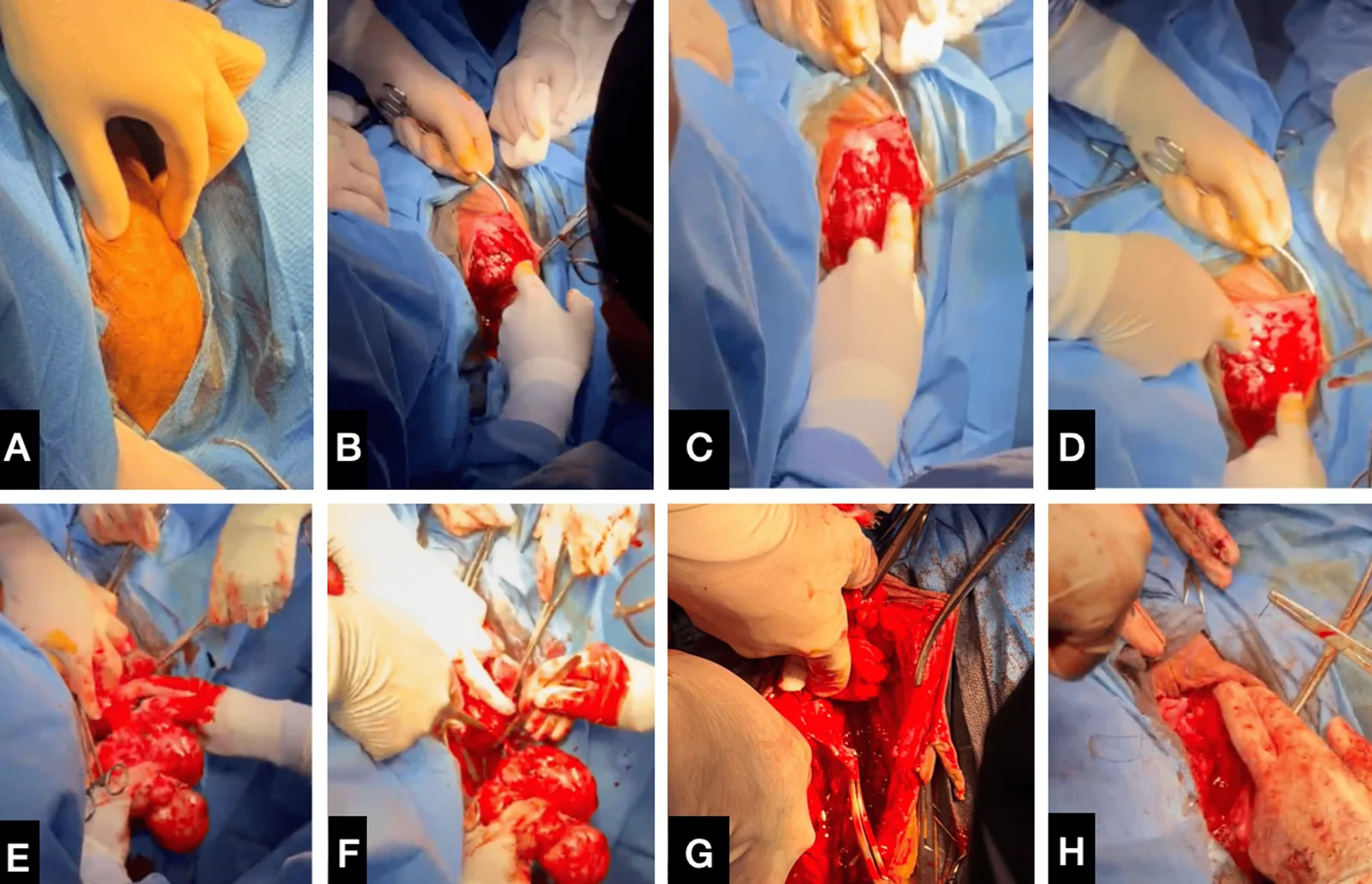
Stepwise surgical excision of large vulvovaginal tumor (A) Preoperative appearance of the lesion, (B–D) Vertical incision over the vaginal aspect of the mass and identification of the tumor capsule by sharp dissection, (E-F) Progressive mobilization and excision of the lesion using predominantly blunt dissection with ligation of feeding vessels, (H) Residual soft-tissue defect following tumor removal and reconstructive vaginoplasty with layered closure.

The smaller lesion was approached through a separate longitudinal incision measuring approximately 2-3 cm, located on the vaginal wall approximately 2 cm inferior to the urethral meatus. The tumor was grasped with two towel clamps and excised by blunt dissection. Blood loss during this part of the procedure was minimal (Figures 5A-5J). Given the depth of the residual cavity and adequate hemostasis, the defect was left unsutured and packed with a hemostatic sponge. Additional vaginal packing was placed at the completion of the procedure to ensure hemostasis. The postoperative course was uneventful, and the patient was discharged home on postoperative day two without complications. Gross examination of both excised tumors revealed similar firm, fibrous consistency (Figures 6A, 6B). The specimens were submitted for histopathological evaluation. 

**Figure 5:**
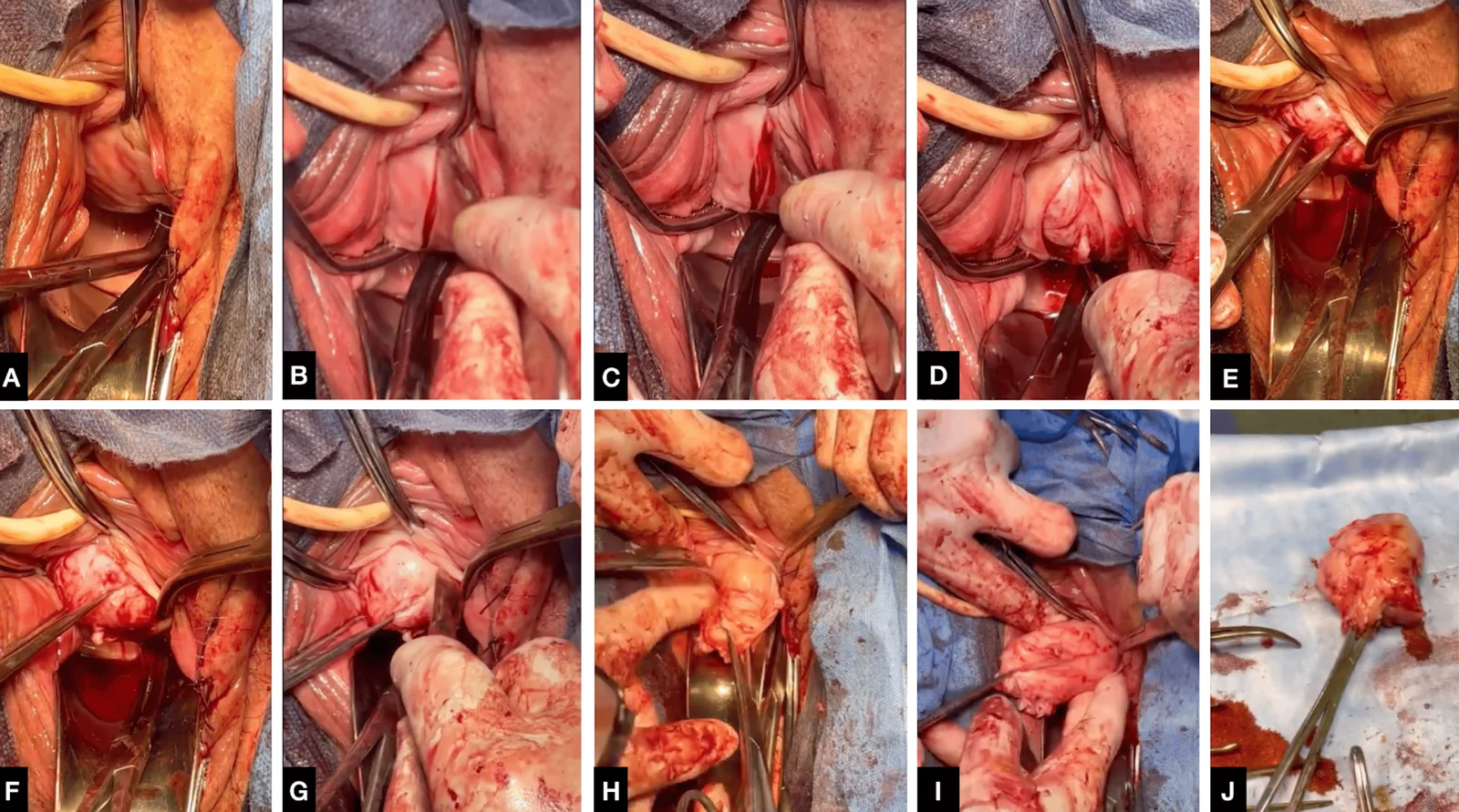
Stepwise surgical excision of a deep vulvar tumor (A) Initial exposure of a partially visible tumor, with a substantial component extending deeply within the soft tissues, (B–D) Careful dissection begins using a scalpel, with progressive capsular separation and mobilization of the lesion from surrounding structures, (E–H) Further blunt and sharp dissection is performed, facilitating gradual mobilization of the mass using a two-toothed forceps for traction and counter-traction, (H-I) Manual assistance is used to aid delivery of the deeply situated tumor from the operative field, (J) Complete excision of the intact mass is achieved, demonstrating the fully removed specimen.

**Figure 6:**
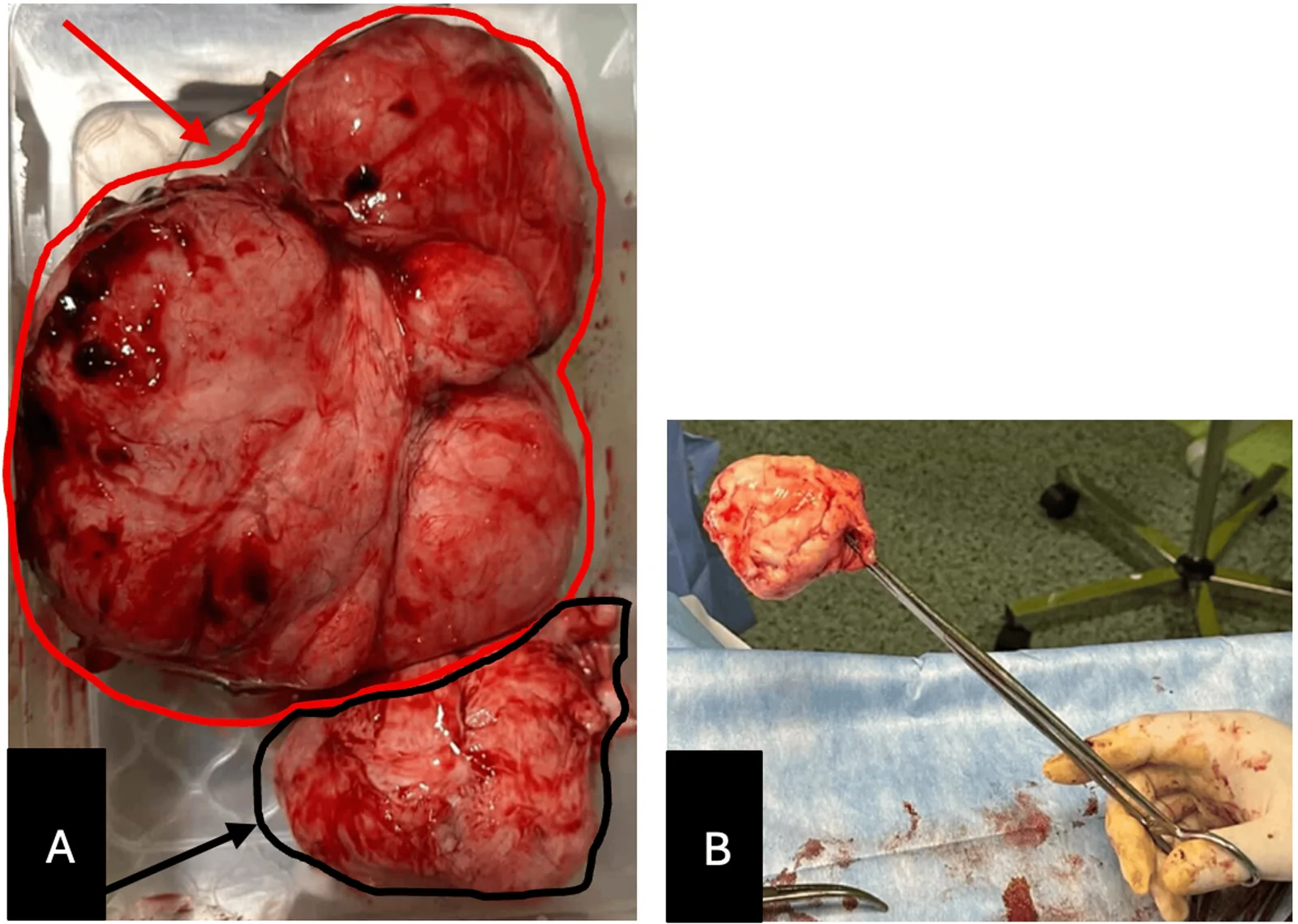
Gross specimen of the excised tumor (A) The red arrow indicates the main, larger mass, which was extensively involved and distorted the left labium major. The black arrow indicates the smaller lesion that was located on the vaginal wall approximately 2 cm inferior to the urethral meatus. (B) Gross appearance of the separately located smaller mass.

Wound care was performed on the patient daily in accordance with the standard protocol. After 2.5 months of treatment, satisfactory wound healing and an improvement in the local condition were observed (Figure* 7*).

**Figure 7:**
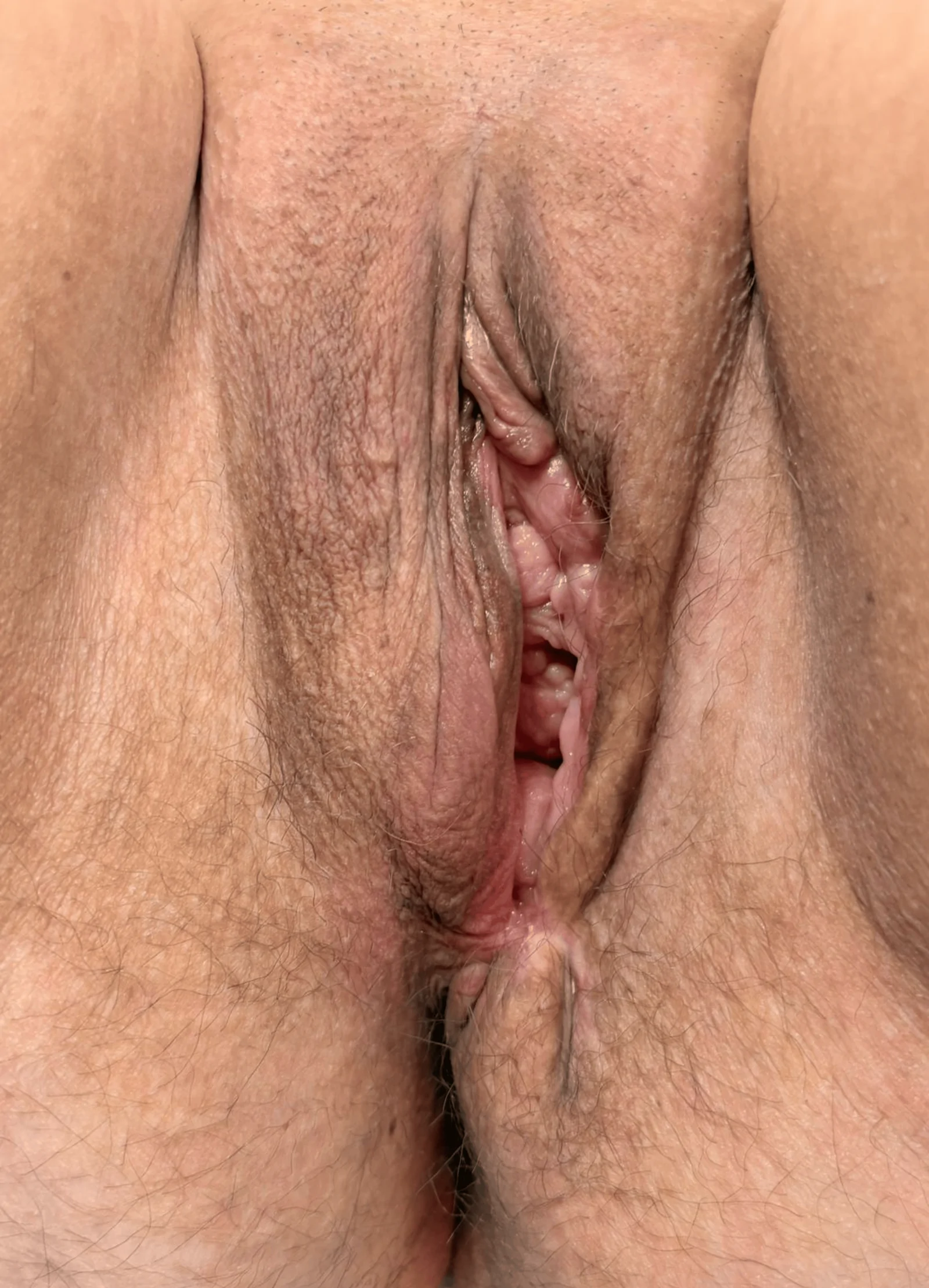
Postoperative appearance of the vulvar region 2.5 months after surgery The wound was managed with daily wound care using Betadine and Microdacyn solution, resulting in satisfactory healing and a favorable postoperative appearance.

Histological and immunohistochemical study

A mesenchymal-derived squamous cell tumor was detected in the tissues of the vulva and vagina, which was characterized by increased cellularity, signs of smooth muscle differentiation, without cytological atypia, pronounced mitotic activity, and tumor necrosis (Figures 8A, 8B).

**Figure 8:**
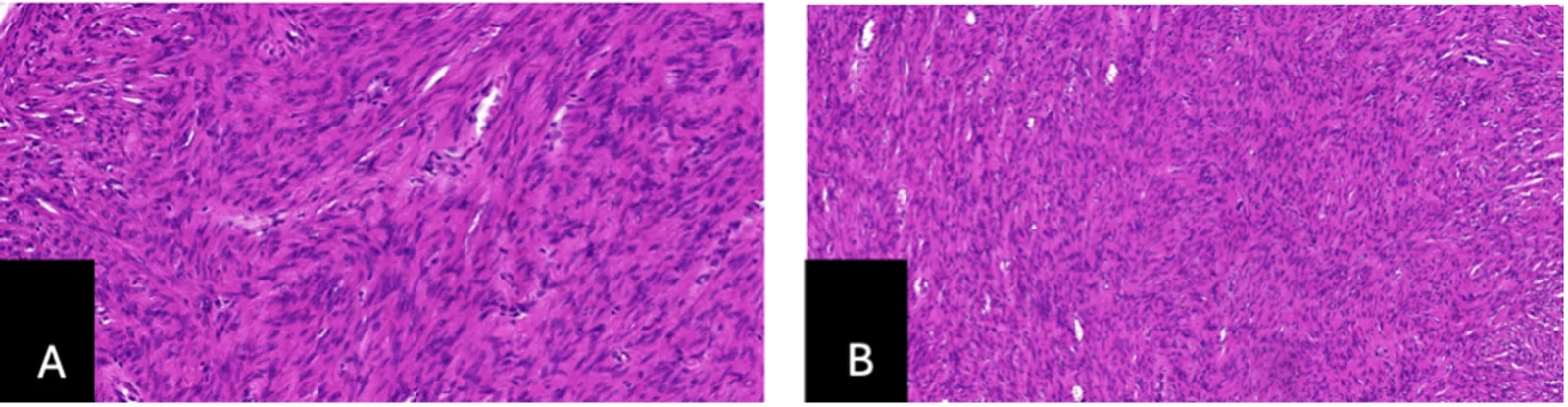
Microscopic images: hematoxylin and eosin staining Histopathological findings at 10× (A) and 20× (B) magnification, demonstrating the nodular structure formed by zones consisting of bundles of oval and spindle-shaped cells arranged both parallel and perpendicular to one another. The nodular structure is covered by stratified squamous epithelium containing foci of mitotic activity. An extensive area of ​​hemorrhage is observed; it is infiltrated by hemosiderophages and bordered peripherally by fibrous and hyaline fibers. Within the subepithelial component, a single focus of mucin-secreting glandular elements is visualized.

On immunohistochemical study, tumor cells expressed SMA (smooth muscle actin), desmin, and h-caldesmon markers, which indicated smooth muscle differentiation. The Ki-67 proliferation index was low (5%), which was consistent with the benign nature of the formation (Figures 9A-9E) (Table 1). Based on morphological and immunohistochemical data, a diagnosis of cellular leiomyoma was established.

**Figure 9:**
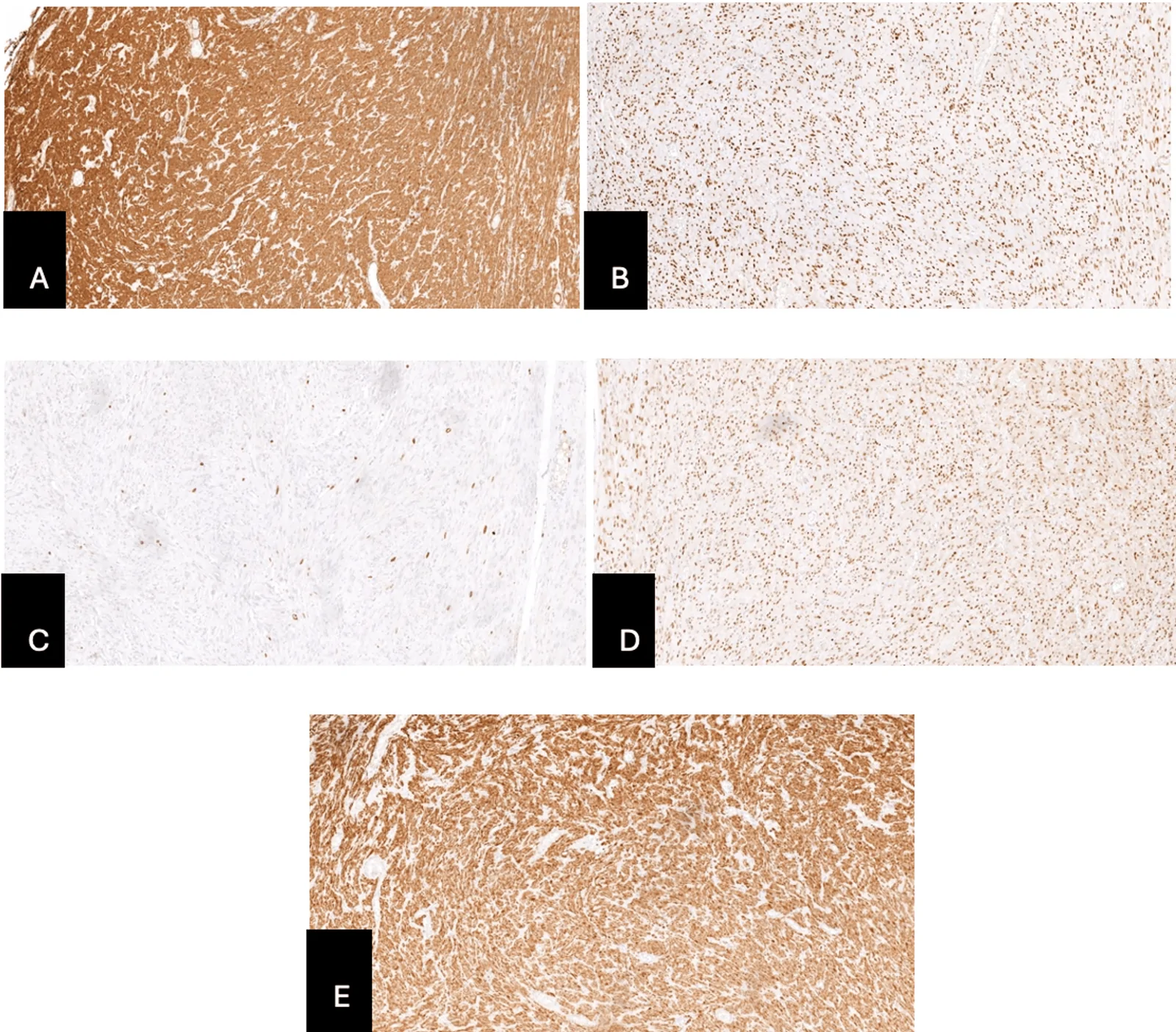
Microscopic findings: immunohistochemistry (IHC) Microscopic images of immunohistological staining for (A) SMA (magnification, x10), (B) PR (magnification, x10), (C) Ki-67 (magnification, x10), (D) ER (magnification, x10) and (E) DESMIN (magnification, x10).

**Table 1:** The immunohistochemical profile The immunohistochemical profile supports a diagnosis of vulvovaginal cellular leiomyoma, confirming smooth muscle differentiation and helping exclude important differential diagnoses, including leiomyosarcoma and other mesenchymal tumors.

Immunohistochemical marker	Result	Significance
SMA	Positive (+)	Confirms smooth muscle differentiation.
Desmin	Positive (+)	Supports smooth muscle origin.
h-Caldesmon	Positive (+)	Specific marker for smooth muscle neoplasm.
CD34	Negative (−)	Helps exclude cellular angiofibroma and other mesenchymal tumors.
S100	Negative (−)	Helps exclude neural tumors.
Ki-67	Low	Supports low proliferative activity and benign behavior.

## Discussion

Vulvovaginal cellular leiomyoma is a more cellular variant of common leiomyoma and rarely develops extrauterinely in the soft tissues of the vulva or vaginal wall. Clinically, it most often presents as a single, slow-growing, and painless mass. Larger masses may cause local symptoms, including discomfort when sitting, walking, having intercourse, or urinating.

Vulvovaginal mesenchymal tumors are a rare and heterogeneous group that includes cellular leiomyoma, angiomyofibroblastoma, cellular angiofibroma, superficial myofibroblastoma, aggressive angiomyxoma, and others. Their diagnosis is often difficult because there is significant morphological and immunohistochemical overlap between the different types [3-5]. Mesenchymal tumors of the vulva and vagina are mainly found in women of reproductive age and often present as slow-growing, painless soft tissue masses. However, large vulvar masses require careful evaluation, as they may clinically resemble malignant tumors and diagnoses such as sarcoma must be excluded [5]. 

In the present case, the presence of two large, long-term progressive, independent masses, which involved both the vulva and the anterior vaginal wall, was a particular challenge. Such a clinical picture requires the exclusion of malignant tumors, including sarcoma. However, preoperative magnetic resonance imaging (MRI) did not reveal invasion of adjacent organs, and histopathological evaluation confirmed a benign mesenchymal mass without cytological atypia. Cellular leiomyoma is a rare histological variant of leiomyoma, characterized by increased cellularity but lacking the major criteria for malignancy, including marked cytological atypia and tumor necrosis. Its differentiation from leiomyosarcoma and other vulvovaginal stromal tumors is important for determining the correct treatment strategy. Immunohistochemical studies play an important role in the diagnostic process. Markers of smooth muscle differentiation, such as SMA, desmin, and h-caldesmon, help confirm cellular leiomyoma and differentiate it from other mesenchymal tumors [3,4].

In the case of benign vulvar leiomyoma, complete surgical excision is the treatment of choice and is usually curative. Complete excision of the tumor with free surgical margins is important to reduce the risk of local recurrence [3,6]. In the case of smooth muscle tumors of uncertain malignant potential (STUMP), complete resection with negative margins and long-term clinical observation is recommended; some authors even consider margins of at least 10 mm. In the case of a diagnosis of leiomyosarcoma, wide local excision is required, and additional treatment - chemotherapy, radiotherapy, or, in selected cases, hormonal therapy, including GnRH analogs is determined on an individual basis. In the presence of morphological or immunohistochemical signs characteristic of fumarate hydratase (FH) deficiency, genetic counseling is recommended [3].

The mainstay of management of vulvovaginal leiomyomas, including cellular leiomyomas, is complete surgical excision. In most cases described in the literature, complete tumor removal is associated with a good prognosis and a low risk of recurrence [3,6]. Surgical treatment of large vulvovaginal masses presents a particular challenge, as the goal is not only to completely remove the tumor, but also to maximally preserve anatomical structures and improve postoperative functional and aesthetic outcomes [7,8]. In contrast to small lesions, wide excision can result in a significant soft tissue defect, which requires an individualized reconstructive approach.

In the present case, due to the large size and vulvovaginal location of the tumor, complete excision was combined with reconstructive vaginoplasty, which allowed the restoration of anatomical integrity and the achievement of a satisfactory functional and aesthetic outcome. Such an approach is especially important in young patients, where the goal of treatment should include not only disease control, but also maintenance of quality of life [9,10].

Therefore, in the case of large vulvovaginal masses, a multidisciplinary approach is necessary, combining clinical, radiological, and histopathological evaluation. Benign mesenchymal tumors, including cellular leiomyomas, should always be considered in the differential diagnosis so that the patient receives individually selected, effective, and less invasive treatment.

## Conclusions

Large vulvovaginal masses in young patients require a comprehensive diagnostic approach to exclude malignancy and to determine the optimal treatment strategy, taking into account the patient's health, quality of life, and organ function. In rare mesenchymal tumors, a definitive morphological diagnosis is essential before treatment, as accurate diagnosis helps to avoid unnecessary radical surgical intervention.

The presented case highlights the importance of integrating clinical, radiological, and histopathological data in the evaluation of vulvovaginal mesenchymal tumors. Benign mesenchymal masses, including cellular leiomyomas, should be considered in the differential diagnosis of vulvovaginal masses. An individually selected surgical approach and, if necessary, the use of reconstructive techniques allow the achievement of good functional and aesthetic results while maintaining the patient's quality of life.
